# Association between glucosamine use and cancer mortality: A large prospective cohort study

**DOI:** 10.3389/fnut.2022.947818

**Published:** 2022-11-02

**Authors:** Jian Zhou, Ziyi Wu, Zhengjun Lin, Wanchun Wang, Rongjun Wan, Tang Liu

**Affiliations:** ^1^Department of Orthopedics, The Second Xiangya Hospital of Central South University, Changsha, Hunan, China; ^2^Department of Respiratory Medicine, National Key Clinical Specialty, Branch of National Clinical Research Center for Respiratory Disease, Xiangya Hospital, Central South University, Changsha, Hunan, China; ^3^Laboratory of Bone Disorder, The Second Xiangya Hospital of Central South University, Changsha, Hunan, China

**Keywords:** glucosamine, cancer, cohort study, mortality, UK Biobank

## Abstract

**Objective:**

Previous studies have shown anti-cancer and anti-inflammatory benefits of glucosamine. This study was performed to prospectively evaluate the association between glucosamine supplementation and the mortality of multiple cancers based on the UK Biobank cohort study.

**Materials and methods:**

A total of 453,645 participants aged 38–73 who had no cancer at baseline were recruited between 2006 and 2010 and followed until March 2021. We used cox and poission proportional hazards models to explore the association between habitual use of glucosamine and cancer mortality. Subgroup analyses were conducted to understand the potential effect modifications of demographics, lifestyle factors, and health outcomes. Sensitivity analyses were performed to determine the robustness of the results.

**Results:**

Of the participants, 88,224 (19.4%) reported habitual glucosamine use at baseline. There were 9,366 cancer deaths during a median follow-up of 12.1 years, and we observed a significant association between the use of glucosamine and lower overall cancer mortality (HR = 0.95, 95% CI = 0.90–1.00, *p* < 0.05), kidney cancer (IRR = 0.68, 95% CI = 0.49–0.95, *p* < 0.05), lung cancer mortality (IRR = 0.84, 95% CI = 0.74–0.95, *p* < 0.05), and rectum cancer (IRR = 0.76, 95% CI = 0.59–0.98, *p* < 0.05). Subgroup analysis showed that habitual glucosamine supplementation was correlated with lower overall cancer mortality among participants who were aged ≥ 60 years, male, current smoker, without high cholesterol and not obese. Sensitivity analysis showed that the results were stable.

**Conclusion:**

Habitual glucosamine use was significantly related to decreased overall cancer, kidney cancer, lung cancer, and rectum cancer mortality, based on data from the large-scale, nationwide, prospective UK Biobank cohort study.

## Introduction

As a non-mineral and non-vitamin supplement, glucosamine is only available by prescription in European countries. However, in Australia and the United States, glucosamine is available over the counter (1, 2). About one in five adults in Australia and 2.6% adults in the United States regularly take glucosamine supplements (3, 4). Glucosamine is an important component in the synthesis of proteoglycans in the human articular cartilage matrix and is widely used in the treatment of osteoarthritis (5). Additionally, glucosamine can regulate various signaling pathways and play a pharmacological role in multiple diseases, including skin diseases, cancer, bacterial infections, and cardiovascular diseases (1, 6, 7). A previous study showed that glucosamine could inhibit the proliferation of a range of tumor cells by inducing cell cycle arrest and apoptosis in cancer cells (8). Some *in vitro* and *in vivo* studies have shown that glucosamine reduces the production of pro-inflammatory factors by inhibiting the mRNA transcription and/or protein expression of pro-inflammatory factors, thereby exerting anti-inflammatory and tumor suppressor effects (9). Moreover, glucosamine can regulate the activity of various important transcription factors and affect various signal transduction pathways, thereby exerting anti-tumor effects. Additionally, according to a large, prospective cohort study, regular use of glucosamine was related to decreased cancer mortality (10). Furthermore, regular glucosamine supplementation was associated with lower lung cancer mortality in the UK Biobank cohort (11) and this work was conducted to expand the evaluation to all cancers. Currently, evidence on the relationship between glucosamine and different types of cancers remained limited.

Therefore, in the present study, we aimed to understand the association of habitual glucosamine use and cancer mortality using the UK Biobank data. Additionally, the potential effect modifications of certain cancer risk factors were explored.

## Materials and methods

### Study population

The UK Biobank is one of the largest population studies in the world aimed at improving the prevention, diagnosis, and treatment of various diseases as well as promoting health across society. The UK Biobank data is open and has been used by researchers around the world (12–16). Over half a million participants aged 40–70 from across the UK were included in the UK Biobank between 2006 and 2010. Participants provided detailed self-reported data at baseline through touchscreen questionnaires and oral interviews with trained nurses at the assessment center. Extensive body measurements were also collected.

**FIGURE 1:**
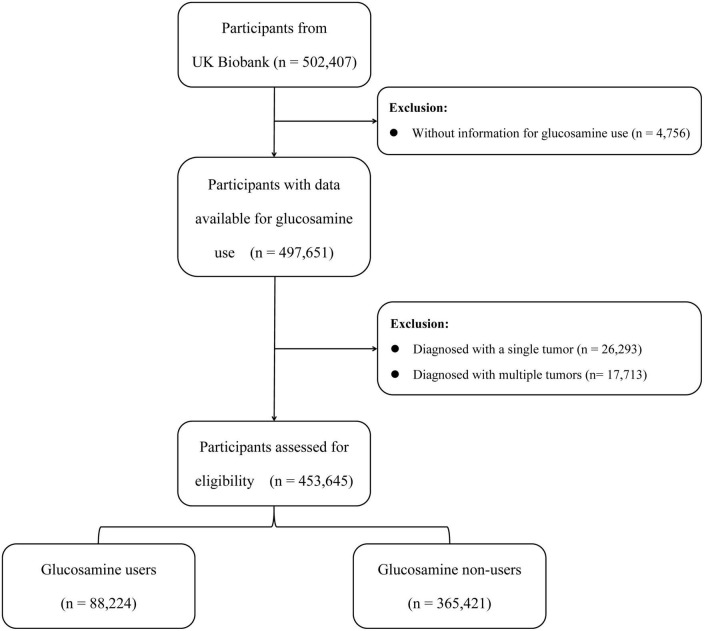
Flowchart of the participants selection.

In this study, a total of 502,407 participants were recruited from the UK Biobank. Participants without information on the use of glucosamine were excluded (*n* = 4,756). Additionally, we excluded participants with one cancer diagnosis at baseline (*n* = 26,293) as well as those with multiple cancer diagnoses (*n* = 17,713). Ultimately, a total of 453,645 participants aged 38–73 were included in this study (Figure 1). Written consent was obtained from all participants, and the UK Biobank study was approved by the North West Multi-centre Research Ethics Committee in the United Kingdom.

### Glucosamine use and measurement of outcome

Participants were asked whether they regularly took supplements including glucosamine at baseline, and those with “yes” responses were defined as habitual glucosamine users. Incidence and survival time data for tumors and deaths was obtained through links to national registries, where cancer cases were classified *via* the International Classification of Diseases, 10th Revision (ICD-10) codes. Self-reported cancer cases were also validated through interviews with trained nurses. Details on tumor mortality validation are available at https://biobank.ctsu.ox.ac.uk/showcase/label.cgi?id=2000. Participants were followed from baseline until death or 1 March 2021, whichever came first.

### Other variables

Variables of interest were noted in this study, including age (in years), gender, ethnic background, average total household income, obesity, physical activity, smoking status, use of alcohol, fruit and vegetable intake, processed meat and red meat intake, supplementation, and drug use (minerals, aspirin, NSAIDS, chondroitin and vitamin). We calculated the body mass index (BMI) as the weight in kilograms (kg) divided by the square of the height in meters (m^2^) and the obesity was defined as BMI ≥ 30 kg/m^2^. According to healthy physical activity recommendations (17), we categorized the enrolled participants into two groups based on the total time spent in moderate physical activity in minutes: <150 or ≥150 min/week.

### Statistical analysis

All the missing covariate values were imputed using multiple imputation with chained equations. Lilliefors tests were conducted to detect whether the data were normally distributed. Continuous variables were indicated as mean ± standard deviation (SD) for normal distributions and median and IQR for non-normal distributions. Categorical variables were indicated as counts and percentages. Cox proportional hazards models were adopted to show the correlation between glucosamine use and overall cancer mortality using hazard ratios (HR) and 95%. Poission proportional hazards models were used to explore the association of glucosamine use and multiple cancers mortality using incidence rate ratio (IRR) and 95% CI. We constructed two models, the basic model and adjusted model, to evaluate the connection between regular use of glucosamine and cancer mortality. The basic model was adjusted by age (years), gender (male or female), ethnic background (white or others), and average total household income (<£18,000, £18,000–£30,999, £31,000–£51,999, £52,000–£100,000, or >£100,000) to analyze the association between glucosamine use and cancer mortality. The adjusted model was also further adjusted for obesity (BMI < 30 or BMI ≥ 30), physical activity (<150 min/week or ≥150 min/week), current smoking (yes or no), alcohol intake (<1, 1–2, 3–4, or >4 times/week), minerals supplementation (calcium, zinc, iron, and selenium) (yes or no), fruit intake (<2.0, 2.0–3.9, or ≥4.0 pieces/day), vegetable intake (<2.0, 2.0–3.9, or ≥4.0 tablespoons/day), processed meat intake (0, 0–1, or >1 times/week), red meat intake (0, 0–1, or >1 times/week), aspirin use (yes or no), NSAIDS use (yes or no), chondroitin use (yes or no), and vitamin use (yes or no). Li et al. evaluated pack years in this population previously and found that it did not alter the association between glucosamine and lung cancer (11); therefore, we did not include pack year for analysis in this study. All the results were indicated as HR/IRR, 95% CI, and *P*-values.

To reveal the potential effect modifications on the association of habitual glucosamine use and cancer mortality, we conducted several subgroup analyses by age (<60 vs. ≥60 years), ethnic background (white vs. others), gender (males vs. females), smoking (yes vs. no), diabetes (yes vs. no), high cholesterol (yes vs. no), arthritis (yes vs. no), and obesity (yes vs. no).

Additionally, we performed a series of sensitivity analyses to reveal the robustness of our results. Participants with glucosamine supplementation also tended to take other supplements more often than participants without glucosamine supplementation. Therefore, we performed sensitivity analyses by removing participants who used other supplementation. Additionally, we removed these participants with missing data to observe the robustness of our results. R version 4.1.2^1^ was adopted for analysis in the present study and two-sided *P*-values of <0.05 were considered statistically significant.

## Results

### Features of participants

A total of 453,645 participants aged 38–73 were enrolled between 2006 and 2010 and followed up until March 2021. Of the participants, 88,224 reported taking glucosamine supplements habitually, while 365,421 reported no history of regular glucosamine supplementation. The median age of all participants was 57.00 years and 54.2% were female. Additionally, 94% of the participants were white. Detailed participant features at baseline are shown in Table 1. Participants with regular glucosamine supplementation were older and more likely to be female compared with non-glucosamine users. In addition, they were more likely to consume minerals, fish oil, and vitamins (such as vitamins A, B, C, D, E, and B9) than participants without habitual glucosamine supplementation.

**TABLE 1:** Baseline features for UK Biobank participants by glucosamine use.

Characteristics	Overall	Glucosamine non-user	Glucosamine user
No. of participants	453,645 (100%)	365,421 (80.6%)	88224 (19.4%)
Age (median [IQR])	57.00 [50.00, 63.00]	56.00 [49.00, 62.00]	60.00 [54.00, 64.00]
Female (%)	245,726 (54.2)	190,906 (52.2)	54,820 (62.1)
**Ethnic background (%)**			
Others	27,058 (6.0)	22,920 (6.3)	4,138 (4.7)
White	426,587 (94.0)	342,501 (93.7)	84,086 (95.3)
**Average total household income (£)**			
<18,000	104,696 (23.1)	85,619 (23.4)	19,077 (21.6)
18,000–30,999	116,336 (25.6)	91,256 (25.0)	25,080 (28.4)
31,000–51,999	117,769 (26.0)	94,472 (25.9)	23,297 (26.4)
52,000–100,000	90,343 (19.9)	73,781 (20.2)	16,562 (18.8)
>100,000	24,501 (5.4)	20,293 (5.6)	4,208 (4.8)
Obesity (%)	111,030 (24.5)	90,483 (24.8)	20,547 (23.3)
**Physical activity (Min/Week)**			
<150	184,836 (40.7)	153,841 (42.1)	30,995 (35.1)
≥150	268,809 (59.3)	211,580 (57.9)	57,229 (64.9)
Current smoking (%)	48,396 (10.7)	42,595 (11.7)	5,801 (6.6)
**Alcohol intake (times/week)**			
<1	139,549 (30.7)	115,251 (31.5)	24,298 (27.5)
1–2	117,466 (25.9)	95,206 (26.1)	22,260 (25.2)
3–4	105,022 (23.2)	82,919 (22.7)	22,103 (25.1)
>4	91,608 (20.2)	72,045 (19.7)	19,563 (22.2)
Minerals supplementation (%)	60,222 (13.3)	38,283 (10.5)	21,939 (24.9)
**Fruit intake (pieces/day)**			
<2.0	161,936 (35.7)	137,628 (37.7)	24,308 (27.6)
2.0–3.9	218,450 (48.2)	171,995 (47.1)	46,455 (52.7)
≥4.0	73,259 (16.1)	55,798 (15.3)	17,461 (19.8)
**Vegetable intake (tablespoons/day)**			
<2.0	28,455 (6.3)	25,084 (6.9)	3,371 (3.8)
2.0–3.9	129,515 (28.5)	107,350 (29.4)	22,165 (25.1)
≥4.0	295,675 (65.2)	232,987 (63.8)	62,688 (71.1)
**Processed meat intake (times/week)**			
0	42,600 (9.4)	33,712 (9.2)	8,888 (10.1)
0–1	137,779 (30.4)	107,724 (29.5)	30,055 (34.1)
>1	273,266 (60.2)	223,985 (61.3)	49,281 (55.9)
**Pork intake (times/week)**			
0	79,298 (17.5)	64,848 (17.7)	14,450 (16.4)
0–1	257,310 (56.7)	205,402 (56.2)	51,908 (58.8)
>1	117,037 (25.8)	95,171 (26.0)	21,866 (24.8)
**Lamb mutton intake (times/week)**			
0	81,655 (18.0)	66,771 (18.3)	14,884 (16.9)
0–1	256,877 (56.6)	205,673 (56.3)	51,204 (58.0)
>1	115,113 (25.4)	92,977 (25.4)	22,136 (25.1)
**Beef intake (times/week)**			
0	51,003 (11.2)	41,338 (11.3)	9,665 (11.0)
0–1	206,216 (45.5)	164,433 (45.0)	41,783 (47.4)
>1	196,426 (43.3)	159,650 (43.7)	36,776 (41.7)
**Poultry intake (times/week)**			
0	23,599 (5.2)	19,241 (5.3)	4,358 (4.9)
0–1	48,508 (10.7)	39,445 (10.8)	9,063 (10.3)
>1	381,538 (84.1)	306,735 (83.9)	74,803 (84.8)
Aspirin use (%)	61,956 (13.7)	50,414 (13.8)	11,542 (13.1)
NSAIDS use (%)	52,813 (11.6)	43,898 (12.0)	8,915 (10.1)
Chondroitin use (%)	4,491 (1.0)	1,016 (0.3)	3,475 (3.9)
Vitamin use (%)	78,022 (17.2)	52,767 (14.4)	25,255 (28.6)

### Relationship between glucosamine supplementation and cancer mortality

**FIGURE 2:**
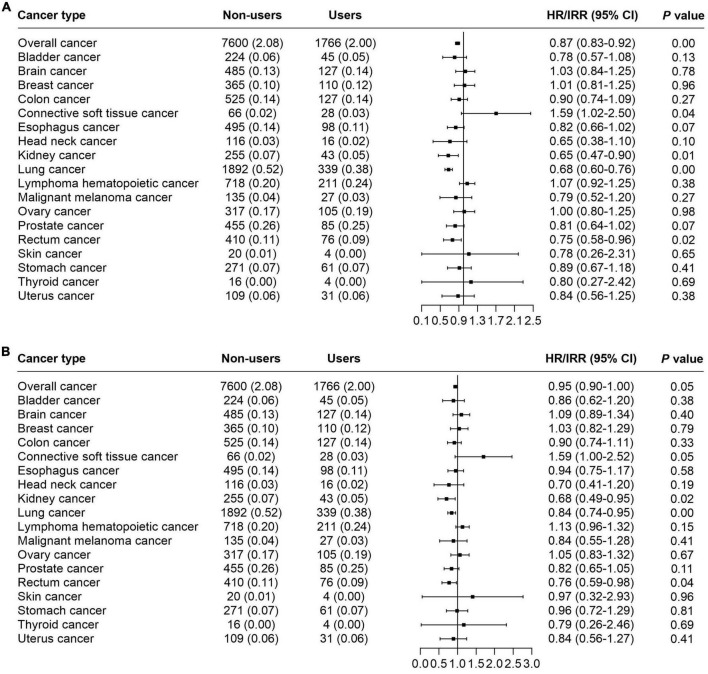
The forest plot indicating the correlation of habitual use of glucosamine and cancer mortality. **(A)** Basic model adjusted by age, gender, ethnic, and average total household. **(B)** Adjusted model performed *via* adjusting of age, gender, ethnic background, average total household income, obesity, physical activity, current smoking, alcohol intake, minerals supplementation, fruit intake, vegetable intake, processed meat intake, red meat intake, aspirin use, NSAIDS use, chondroitin use, and vitamin use.

According to the results, we found that glucosamine use was significantly associated with decreased mortality in overall cancer (HR = 0.87, 95% CI = 0.83–0.92, *p* < 0.05), kidney cancer (IRR = 0.65, 95% CI = 0.47–0.90, *p* < 0.05), lung cancer (IRR = 0.68, 95% CI = 0.60–0.76, *p* < 0.05), rectum cancer (IRR = 0.75, 95% CI = 0.58–0.96, *p* < 0.05). These results were obtained from the basic model with age, gender, ethnic background, and average total household income adjusted (Figure 2A and Table 2). The model was then further adjusted by obesity, physical activity, current smoking, alcohol intake, minerals supplementation, fruit intake, vegetable intake, processed meat intake, red meat intake, aspirin use, NSAIDS use, chondroitin use, and vitamin use. With this model, we noted that supplementation of glucosamine was related to lower mortality for overall cancer (HR = 0.95, 95% CI = 0.90–1.00, *p* < 0.05), kidney cancer (IRR = 0.68, 95% CI = 0.49–0.95, *p* < 0.05), lung cancer mortality (IRR = 0.84, 95% CI = 0.74–0.95, *p* < 0.05), and rectum cancer (IRR = 0.76, 95% CI = 0.59–0.98, *p* < 0.05) (Figure 2B and Table 2).

**TABLE 2:** Associations of glucosamine supplement use with cancer mortality.

Cancer type	Glucosamine non-users	Glucosamine users	Basic model*		Adjusted model^#^	
			HR/IRR (95% CI)	*P-*value	HR/IRR (95% CI)	*P-*value
Overall cancer	7,600 (2.08)	1,766 (2.00)	0.87 (0.83–0.92)	<0.01	0.95 (0.90–1.00)	0.05
Bladder cancer	224 (0.06)	45 (0.05)	0.78 (0.57–1.08)	0.13	0.86 (0.62–1.20)	0.38
Brain cancer	485 (0.13)	127 (0.14)	1.03 (0.84–1.25)	0.78	1.09 (0.89–1.34)	0.40
Breast cancer	365 (0.10)	110 (0.12)	1.01 (0.81–1.25)	0.96	1.03 (0.82–1.29)	0.79
Colon cancer	525 (0.14)	127 (0.14)	0.90 (0.74–1.09)	0.27	0.90 (0.74–1.11)	0.33
Connective softTissue cancer	66 (0.02)	28 (0.03)	1.59 (1.02–2.50)	0.04	1.59 (1.00–2.52)	0.05
Esophagus cancer	495 (0.14)	98 (0.11)	0.82 (0.66–1.02)	0.07	0.94 (0.75–1.17)	0.58
HeadNeck cancer	116 (0.03)	16 (0.02)	0.65 (0.38–1.10)	0.10	0.70 (0.41–1.20)	0.19
Kidney cancer	255 (0.07)	43 (0.05)	0.65 (0.47–0.90)	0.01	0.68 (0.49–0.95)	0.02
Lung cancer	1,892 (0.52)	339 (0.38)	0.68 (0.60–0.76)	<0.01	0.84 (0.74–0.95)	0.00
Lymphoma hematopoietic cancer	718 (0.20)	211 (0.24)	1.07 (0.92–1.25)	0.38	1.13 (0.96–1.32)	0.15
Malignant melanoma cancer	135 (0.04)	27 (0.03)	0.79 (0.52–1.20)	0.27	0.84 (0.55–1.28)	0.41
Ovary cancer	317 (0.17)	105 (0.19)	1.00 (0.80–1.25)	0.98	1.05 (0.83–1.32)	0.67
Prostate cancer	455 (0.26)	85 (0.25)	0.81 (0.64–1.02)	0.07	0.82 (0.65–1.05)	0.11
Rectum cancer	410 (0.11)	76 (0.09)	0.75 (0.58–0.96)	0.02	0.76 (0.59–0.98)	0.04
Skin cancer	20 (0.01)	4 (0.00)	0.78 (0.26–2.31)	0.65	0.97 (0.32–2.93)	0.96
Stomach cancer	271 (0.07)	61 (0.07)	0.89 (0.67–1.18)	0.41	0.96 (0.72–1.29)	0.81
Thyroid cancer	16 (0.00)	4 (0.00)	0.80 (0.27–2.42)	0.69	0.79 (0.26–2.46)	0.69
Uterus cancer	109 (0.06)	31 (0.06)	0.84 (0.56–1.25)	0.38	0.84 (0.56–1.27)	0.41

*Basic model: adjusted for age, gender, ethnicity, and average total household income.

^#^Adjusted model: adjusted for age, gender, ethnic background, average total household income, obesity, physical activity, current smoking, alcohol intake, minerals supplementation, fruit intake, vegetable intake, processed meat intake, red meat intake, aspirin use, NSAIDS use, chondroitin use, and vitamin use

### Subgroup analysis

**FIGURE 3:**
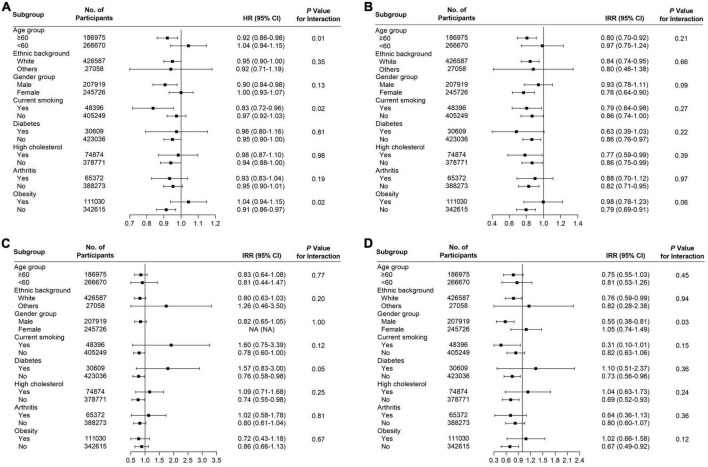
Subgroup analysis for **(A)** all cancers, **(B)** lung cancer, **(C)** prostate cancer, and **(D)** rectum cancer to analyze the potential modification effects between age, ethnic background, gender, smoking, diabetes, high cholesterol, arthritis, and obesity.

**FIGURE 4:**
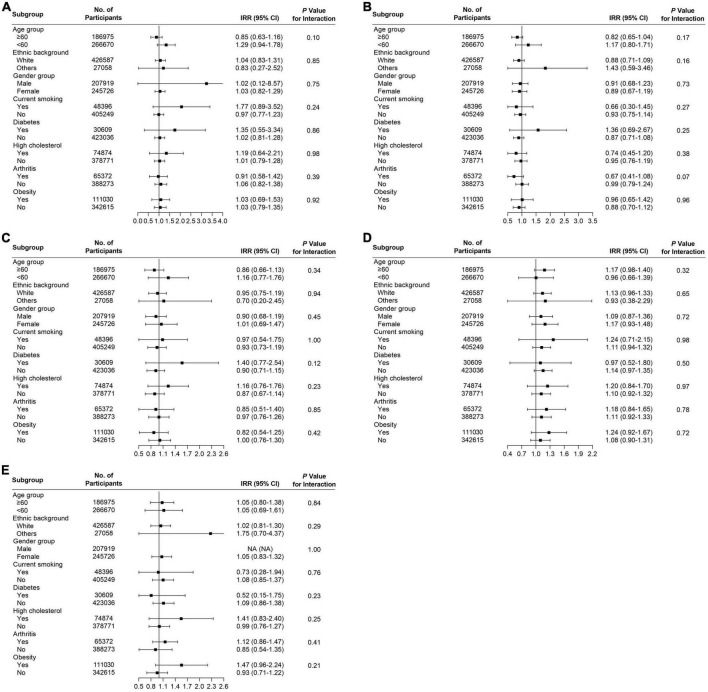
Subgroup analysis for **(A)** breast cancer, **(B)** colon cancer, **(C)** esophagus cancer, **(D)** lymphoma hematopoietic cancer, and **(E)** ovary cancer to analyze the potential modification effects between age, ethnic background, gender, smoking, diabetes, high cholesterol, arthritis, and obesity.

Several subgroup analyses were conducted to analyze the potential effect modifications among the variables of age, ethnicity, gender, smoking, diabetes, high cholesterol, arthritis, and obesity. Significant association between habitual supplementation of glucosamine and lower overall cancer mortality was observed in participants who were aged ≥ 60 years (HR = 0.92, 95% CI = 0.86–0.98, *p* < 0.05), male (HR = 0.90, 95% CI = 0.84–0.98, *p* < 0.05), current smoker (HR = 0.83, 95% CI = 0.72–0.96, *p* < 0.05), without high cholesterol (HR = 0.94, 95% CI = 0.88–1.00, *p* < 0.05), and not obese (HR = 0.90, 95% CI = 0.83–0.98, *p* < 0.05) (Figure 3A). We found that the use of glucosamine was connected to lung cancer in those ≥ 60 years (IRR = 0.80, 95% CI = 0.70–0.92, *p* < 0.05), white (IRR = 0.84, 95% CI = 0.74–0.95, *p* < 0.05), female (IRR = 0.76, 95% CI = 0.64–0.90, *p* < 0.05), current smoker (IRR = 0.79, 95% CI = 0.64–0.98, *p* < 0.05), without diabetes (IRR = 0.86, 95% CI = 0.76–0.97, *p* < 0.05), without arthritis (IRR = 0.82, 95% CI = 0.71–0.95, *p* < 0.05), and not obese (IRR = 0.79, 95% CI = 0.69–0.91, *p* < 0.05) (Figure 3B). Glucosamine supplementation was related to prostate cancer mortality in participants without diabetes (IRR = 0.76, 95% CI = 0.58–0.98, *p* < 0.05) and without high cholesterol (IRR = 0.74, 95% CI = 0.55–0.98, *p* < 0.05) (Figure 3C) and rectum cancer mortality in participants who were white (IRR = 0.76, 95% CI = 0.59–0.99, *p* < 0.05), males (IRR = 0.55, 95% CI = 0.38–0.81, *p* < 0.05), without diabetes (IRR = 0.73, 95% CI = 0.56–0.96, *p* < 0.05), without high cholesterol (IRR = 0.69, 95% CI = 0.52–0.93, *p* < 0.05), and not obese (IRR = 0.67, 95% CI = 0.49–0.92, *p* < 0.05) (Figure 3D). The association of glucosamine use with the overall cancer mortality was stronger among participants who were more than 60 years (*P* for interaction = 0.01) current smoker (*P* for interaction = 0.02) and not obese (*P* for interaction = 0.02) (Figure 3). The connection between glucosamine use and the rectum cancer mortality was stronger among participants who were male (*P* for interaction = 0.03) (Figure 3). No more finding was observed from subgroup analysis of breast cancer (Figure 4A), colon cancer (Figure 4B), esophagus cancer (Figure 4C), lymphoma hematopoietic cancer (Figure 4D), and ovary cancer (Figure 4E).

### Sensitivity analysis

The correlation between glucosamine supplementation and cancer mortality did not change substantially after we removed participants who took other supplements (Supplementary Table 1). Moreover, when we removed the participants with missing values for covariates, the conclusion were unchanged (Supplementary Table 2). The results from our sensitivity analysis indicated that the results we obtained were stable.

## Discussion

Glucosamine is an amino sugar substance formed by replacing one hydroxyl group of glucose with an amino group. Endogenous glucosamine is synthesized *in vivo* through the hexosamine biosynthesis pathway (HBP), and it occurs naturally in most human tissues, with the highest levels being in healthy cartilage (18). Glucosamine is one of the most commonly taken dietary supplements in Australia, and it is commonly used to treat rheumatoid arthritis and osteoarthritis. The substance can regulate a variety of signaling pathways and play pharmacological roles in various diseases. However, the impact of glucosamine supplementation on cancer mortality remains unclear. Here we conducted a large prospective cohort study with more than 450,000 participants enrolled from the UK Biobank. We observed that glucosamine supplementation was correlated with a 5% lower risk of overall cancer mortality, 32% lower risk of kidney cancer mortality, 16% lower risk of lung cancer, and 24% lower risk of rectum cancer mortality. Notably, the association between glucosamine use and lung cancer mortality had previously been evaluated in this cohort with similar results (11).

According to the research progress *in vitro* and *in vivo*, glucosamine can interact with multiple molecular targets, regulate multiple cell signaling pathways, and have great therapeutic potential for various cancers (19). The anti-tumor effects of glucosamine are mainly achieved by inhibiting the proliferation of cancer cells and inducing apoptosis, inducing autophagic death of cancer cells, reversing tumor drug resistance, anti-tumor angiogenesis, and inhibiting the expression of matrix metalloproteinases (20, 21).

Several epidemiologic studies have indicated that glucosamine use is connected to cancer mortality. Bell et al. reported that use of glucosamine was related to a significant decreased risk of death from cancer in the United States (22). Li et al. found that regular use of glucosamine was associated with a lower cancer mortality in the United Kingdom (UK) (10). Additionally, Brasky et al. observed that high 10-year supplementation of glucosamine was related to decreased lung cancer risk in the United States (23). Li et al. found that regular glucosamine supplementation was associated with lower lung cancer mortality in the UK population (11). In the present study, we observed that glucosamine supplementation was associated with a 16% lower risk of lung cancer mortality, which was similar to the findings of Li et al. (11). Compared with Li et al.’s study, we constructed cox and poission proportional hazards models to explore the association between habitual use of glucosamine and multiple cancer mortalities, not merely lung cancer. We found that glucosamine supplementation was also related to a 5% lower risk of overall cancer mortality, 32% lower risk of kidney cancer mortality, 16% lower risk of lung cancer, and 24% lower risk of rectum cancer mortality. Moreover, although we did not find an association between glucosamine use and other cancers among the general population, subgroup analysis indicated that its supplementation was related to a lower risk of mortality for prostate cancer in participants without diabetes/cholesterol and rectum cancer mortality in participants who were white, males, without diabetes, without high cholesterol and not obese.

Several potential mechanisms may contribute to the inverse relationship between habitual use of glucosamine and cancer mortality. Glucosamine reduces the production of pro-inflammatory factors by inhibiting the mRNA transcription and/or protein expression of these pro-inflammatory factors, thereby exerting anti-inflammatory and tumor-suppressive effects (9). Additionally, glucosamine improves the resistance of non-small cell lung cancer cells A549 to TRAIL by upregulating the expression of DR5 (24). Furthermore, glucosamine blocks the VEGF-VEGFR signaling pathway by inhibiting VEGF mRNA expression, inhibiting tumor angiogenesis, and exerting anticancer effects (25). Previous studies confirmed that glucosamine inhibited the proliferation of human non-small cell lung cancer A549 cells and inhibited the expression of downstream transcription factors FoxO1 and FoxO3 (26). Glucosamine also promotes NK cell differentiation through the expression of CD3-CD56 + subsets, promotes T cell differentiation through the expression of CD4 + subsets, induces the secretion of IL-2 and IFN-γ, and activates NK cells and T cells at the same time. Thus, exerting its immune regulation and anti-tumor activity (25).

In addition, subgroup analysis indicated glucosamine use was associated with lower overall cancer mortality among participants who were aged ≥ 60 years, male, current smoker, without high cholesterol and not obese. Further clarification on the mechanisms of this association may be necessary. Furthermore, a significant relationship between glucosamine uses and lower risk of lung cancer mortality was observed in those ≥ 60 years, white, female, current smoker, without diabetes, without arthritis and not obese. The potential explanation for stronger effect against cancer observed among current smokers might be that those smoker are at a state of higher inflammatory stress. Therefore, the anti-inflammatory effect from glucosamine may provide stronger benefit.

Glucosamine and other supplements were often taken together, which we hypothesized may affect the relationship. Therefore, sensitivity analyses were conducted to detect the correlation between glucosamine use alone (excluding participants who took other supplementation) with cancer mortality. We observed that the estimates did not change significantly. In addition, when we excluded the participants with missing values for covariates, the conclusion did not substantially change, making it likely that glucosamine supplementation may decrease cancer mortality regardless of the use of other supplementation or missing values for covariates.

## Strengths and limitations

There were several major strengths of this study, including a minimal loss of follow-up, a large sample size, and a population-based prospective cohort study design. Additionally, the rich information on socioeconomic factors, disease history, and lifestyle allowed us to conduct a comprehensive subgroup analysis. However, there were some limitations in our study. First, there was no detailed information presented on the dose, form, or duration of glucosamine use. Second, although we carefully adjusted for potential confounders related to lifestyle in our analysis, we could not remove the possibility that the results we obtained were confounded by unmeasured factors related to lifestyle. Third, this study evaluated glucosamine use and cancer mortality in the UK. The UK uses a prescription grade formula and the results we obtained may not be generalizable to other populations that use over the counter formulations.

## Conclusion

In conclusion, the present study indicated that regular use of glucosamine supplements was significantly related to decreased overall cancer, kidney cancer, lung cancer, and rectum cancer mortality. Further pharmacological studies are needed to increase our understanding of the potential benefits of glucosamine.

## Data availability statement

This research has been conducted using the UK Biobank resource (https://www.ukbiobank.ac.uk) under application number: 80610.

## Ethics statement

The UK Biobank received ethical approval from the research Ethics Committee (REC reference for UK Biobank 11/NW/0382) and participants provided written informed consent. The patients/participants provided their written informed consent to participate in this study.

## Author contributions

TL designed the study and performed the analysis. JZ and RW drafted the manuscript. ZW, ZL, WW, and TL contributed to the revision of the manuscript. All authors have read and approved the final manuscript.

## Footnotes

1
www.r-project.org

## References

[1] Ma H Li X Sun D Zhou T Ley SH Gustat J Association of habitual glucosamine use with risk of cardiovascular disease: prospective study in UK Biobank. *BMJ.* (2019) 365:l1628. 10.1136/bmj.l1628 PMC6515311 31088786

[2] Ma H Li X Zhou T Sun D Liang Z Li Y Glucosamine use, inflammation, and genetic susceptibility, and incidence of type 2 diabetes: a prospective study in UK Biobank. *Diabetes Care.* (2020) 43:719–25. 10.2337/dc19-1836 31988063 PMC7085804

[3] Sibbritt D Adams J Lui CW Broom A Wardle J. Who uses glucosamine and why? A study of 266,848 Australians aged 45 years and older. *PLoS One.* (2012) 7:e41540. 10.1371/journal.pone.0041540 22859995 PMC3408465

[4] Clarke TC Black LI Stussman BJ Barnes PM Nahin RL. Trends in the use of complementary health approaches among adults: United States, 2002-2012. *Natl Health Stat Rep.* (2015) 79:1–16. PMC4573565 25671660

[5] Jordan KM Arden NK Doherty M Bannwarth B Bijlsma JW Dieppe P Recommendations 2003: an evidence based approach to the management of knee osteoarthritis: report of a Task Force of the Standing Committee for International Clinical Studies Including Therapeutic Trials (ESCISIT). *Ann Rheum Dis.* (2003) 62:1145–55. 10.1136/ard.2003.011742 14644851 PMC1754382

[6] Valinezhad SF Palizban A Mosaffa F Jamialahmadi K. Glucosamine attenuates drug resistance in Mitoxantrone-resistance breast cancer cells. *J Pharm Pharmacol.* (2021) 73:922–7. 10.1093/jpp/rgaa032 33885909

[7] Bissett DL. Glucosamine: an ingredient with skin and other benefits. *J Cosmet Dermatol.* (2006) 5:309–15. 10.1111/j.1473-2165.2006.00277.x 17716251

[8] Wang LS Chen SJ Zhang JF Liu MN Zheng JH Yao XD. Anti-proliferative potential of Glucosamine in renal cancer cells via inducing cell cycle arrest at G0/G1 phase. *BMC Urol.* (2017) 17:38. 10.1186/s12894-017-0221-7 28558682 PMC5450348

[9] Dalirfardouei R Karimi G Jamialahmadi K. Molecular mechanisms and biomedical applications of glucosamine as a potential multifunctional therapeutic agent. *Life Sci.* (2016) 152:21–9. 10.1016/j.lfs.2016.03.028 27012765

[10] Li ZH Gao X Chung VC Zhong WF Fu Q Lv YB Associations of regular glucosamine use with all-cause and cause-specific mortality: a large prospective cohort study. *Ann Rheum Dis.* (2020) 79:829–36. 32253185 10.1136/annrheumdis-2020-217176 PMC7286049

[11] Li G Zhang X Liu Y Zhang J Li L Huang X Relationship between glucosamine use and the risk of lung cancer: data from a nationwide prospective cohort study. *Eur Respir J.* (2022) 59:2101399. 10.1183/13993003.01399-2021 34326189

[12] Li X Zhou T Ma H Huang T Gao X Qi L Healthy sleep patterns and risk of incident arrhythmias. *J Am Coll Cardiol.* (2021) 78:1197–207. 34531019 10.1016/j.jacc.2021.07.023 PMC8454031

[13] Li X Wang M Song Y Ma H Zhou T Liang Z Obesity and the relation between joint exposure to ambient air pollutants and incident type 2 diabetes: a cohort study in UK Biobank. *PLoS Med.* (2021) 18:e1003767. 10.1371/journal.pmed.1003767 34460827 PMC8439461

[14] Li X Zhou T Ma H Liang Z Fonseca VA Qi L. Replacement of sedentary behavior by various daily-life physical activities and structured exercises: genetic risk and incident type 2 diabetes. *Diabetes Care.* (2021) [Online ahead of print]. 10.2337/dc21-0455 34183430 PMC8929188

[15] Li X Xue Q Wang M Zhou T Ma H Heianza Y Adherence to a healthy sleep pattern and incident heart failure: a prospective study of 408 802 UK biobank participants. *Circulation.* (2021) 143:97–9. 10.1161/CIRCULATIONAHA.120.050792 33190528 PMC7775332

[16] Liu D Li ZH Shen D Zhang PD Song WQ Zhang WT Association of sugar-sweetened, artificially sweetened, and unsweetened coffee consumption with all-cause and cause-specific mortality : a large prospective cohort study. *Ann Intern Med.* (2022) 175:909–17. 10.7326/M21-2977 35635846

[17] World Health Organization. *Global Recommendations On Physical Activity For Health.* Geneva: World Health Organization (2010). 26180873

[18] Vasiliadis HS Tsikopoulos K. Glucosamine and chondroitin for the treatment of osteoarthritis. *World J Orthop.* (2017) 8:1–11. 28144573 10.5312/wjo.v8.i1.1 PMC5241539

[19] Masuda S Azuma K Kurozumi S Kiyose M Osaki T Tsuka T Anti-tumor properties of orally administered glucosamine and N-acetyl-D-glucosamine oligomers in a mouse model. *Carbohydr Polym.* (2014) 111:783–7. 10.1016/j.carbpol.2014.04.102 25037416

[20] Jung CW Jo JR Lee SH Park YK Jung NK Song DK Anti-cancer properties of glucosamine-hydrochloride in YD-8 human oral cancer cells: induction of the caspase-dependent apoptosis and down-regulation of HIF-1alpha. *Toxicol In Vitro.* (2012) 26:42–50. 10.1016/j.tiv.2011.10.005 22020377

[21] Pohlig F Ulrich J Lenze U Mühlhofer HM Harrasser N Suren C Glucosamine sulfate suppresses the expression of matrix metalloproteinase-3 in osteosarcoma cells in vitro. *BMC Complement Altern Med.* (2016) 16:313. 10.1186/s12906-016-1315-6 27562075 PMC5000453

[22] Bell GA Kantor ED Lampe JW Shen DD White E. Use of glucosamine and chondroitin in relation to mortality. *Eur J Epidemiol.* (2012) 27:593–603. 22828954 10.1007/s10654-012-9714-6 PMC3557824

[23] Brasky TM Lampe JW Slatore CG White E. Use of glucosamine and chondroitin and lung cancer risk in the VITamins And Lifestyle (VITAL) cohort. *Cancer Causes Control.* (2011) 22:1333–42. 10.1007/s10552-011-9806-8 21706174 PMC3175750

[24] Liang Y Xu W Liu S Chi J Zhang J Sui A Acetyl-glucosamine sensitizes non-small cell lung cancer cells to trail-induced apoptosis by activating death receptor 5. *Cell Physiol Biochem.* (2018) 45:2054–70. 10.1159/000488042 29533936

[25] Xu W Jiang C Kong X Liang Y Rong M Liu W. Chitooligosaccharides and N-acetyl-D-glucosamine stimulate peripheral blood mononuclear cell-mediated antitumor immune responses. *Mol Med Rep.* (2012) 6:385–90. 10.3892/mmr.2012.918 22614871

[26] Yu Z Ju Y Liu H. Antilung cancer effect of glucosamine by suppressing the phosphorylation of FOXO. *Mol Med Rep.* (2017) 16:3395–400. 10.3892/mmr.2017.6976 28713921

