# Supplementary material for: Association between glucosamine use and cancer mortality: A large prospective cohort study
Source: Front Nutr. 2022 Nov 2;9:947818. doi: 10.3389/fnut.2022.947818 (PMC9667031; doi:10.3389/fnut.2022.947818)
Supplement: Supplementary file 1 [file Table_1.doc]

Table S1. Associations between glucosamine and cancer mortality after removing participants who took other supplementation. Values are numbers (%) unless stated otherwise; HR: hazard ratio; IRR: incidence rate ratio; CI: confidence interval.

| Cancer type | Glucosamine Non-Users | Glucosamine Users | Adjusted model^#^ | |
| --- | --- | --- | --- | --- |
|  |  |  | HR/IRR (95% CI) | *P* Value |
| Overall cancer | 7588 (2.08) | 1706 (2.01) | 0.95 (0.90-1.00) | 0.05 |
| Bladder cancer | 224 (0.06) | 45 (0.05) | 0.86 (0.62-1.20) | 0.38 |
| Brain cancer | 485 (0.13) | 125 (0.15) | 1.09 (0.89-1.33) | 0.41 |
| Breast cancer | 364 (0.10) | 107 (0.13) | 1.04 (0.83-1.30) | 0.75 |
| Colon cancer | 523 (0.14) | 119 (0.14) | 0.90 (0.73-1.11) | 0.32 |
| Connective softTissue cancer | 66 (0.02) | 28 (0.03) | 1.59 (1.00-2.52) | 0.05 |
| Esophagus cancer | 494 (0.14) | 98 (0.12) | 0.95 (0.76-1.19) | 0.65 |
| HeadNeck cancer | 116 (0.03) | 16 (0.02) | 0.70 (0.41-1.20) | 0.19 |
| Kidney cancer | 255 (0.07) | 40 (0.05) | 0.67 (0.47-0.94) | 0.02 |
| Lung cancer | 1889 (0.52) | 327 (0.39) | 0.84 (0.74-0.94) | 0.00 |
| Lymphoma hematopoeietic cancer | 718 (0.20) | 201 (0.24) | 1.11 (0.94-1.30) | 0.21 |
| Malignant melanoma cancer | 135 (0.04) | 26 (0.03) | 0.83 (0.54-1.28) | 0.40 |
| Ovary cancer | 317 (0.17) | 100 (0.19) | 1.04 (0.82-1.31) | 0.75 |
| Prostate cancer | 453 (0.26) | 83 (0.26) | 0.83 (0.65-1.06) | 0.13 |
| Rectum cancer | 408 (0.11) | 74 (0.09) | 0.78 (0.60-1.00) | 0.05 |
| Skin cancer | 20 (0.01) | 4 (0.00) | 0.97 (0.32-2.93) | 0.96 |
| Stomach cancer | 271 (0.07) | 58 (0.07) | 0.95 (0.71-1.27) | 0.73 |
| Thyroid cancer | 16 (0.00) | 4 (0.00) | 0.79 (0.26-2.46) | 0.69 |
| Uterus cancer | 109 (0.06) | 30 (0.06) | 0.84 (0.55-1.27) | 0.41 |

^#^ Adjusted model: adjusted for age, gender, ethnic background, average total household income, obesity, physical activity, current smoking, alcohol intake, minerals supplementation, fruit intake, vegetable intake, processed meat intake, red meat intake, aspirin use, NSAIDS use, chondroitin use and vitamin use.

Table S2. Associations between glucosamine and cancer mortality after after excluding participants with missing covariate data. Values are numbers (%) unless stated otherwise; HR: hazard ratio; IRR: incidence rate ratio; CI: confidence interval.

| Cancer type | Glucosamine Non-Users | Glucosamine Users | Adjusted model^#^ | |
| --- | --- | --- | --- | --- |
|  |  |  | HR/IRR (95% CI) | *P* Value |
| Overall cancer | 3857 (2.19) | 1014 (2.01) | 0.94 (0.89-1.00) | 0.05 |
| Bladder cancer | 107 (0.06) | 29 (0.06) | 0.94 (0.66-1.34) | 0.73 |
| Brain cancer | 221 (0.13) | 69 (0.14) | 1.19 (0.94-1.50) | 0.14 |
| Breast cancer | 167 (0.09) | 60 (0.12) | 1.00 (0.77-1.29) | 0.98 |
| Colon cancer | 275 (0.16) | 72 (0.14) | 0.83 (0.66-1.05) | 0.13 |
| Connective softTissue cancer | 38 (0.02) | 17 (0.03) | 1.47 (0.89-2.45) | 0.14 |
| Esophagus cancer | 258 (0.15) | 62 (0.12) | 1.01 (0.80-1.28) | 0.93 |
| HeadNeck cancer | 52 (0.03) | 9 (0.02) | 0.71 (0.38-1.33) | 0.28 |
| Kidney cancer | 143 (0.08) | 29 (0.06) | 0.69 (0.48-0.99) | 0.05 |
| Lung cancer | 949 (0.54) | 206 (0.41) | 0.83 (0.72-0.94) | 0.00 |
| Lymphoma hematopoeietic cancer | 360 (0.20) | 117 (0.23) | 1.12 (0.93-1.34) | 0.22 |
| Malignant melanoma cancer | 65 (0.04) | 15 (0.03) | 0.86 (0.53-1.40) | 0.54 |
| Ovary cancer | 155 (0.17) | 46 (0.15) | 1.02 (0.79-1.32) | 0.89 |
| Prostate cancer | 256 (0.30) | 52 (0.26) | 0.82 (0.62-1.07) | 0.15 |
| Rectum cancer | 208 (0.12) | 41 (0.08) | 0.71 (0.50-1.00) | 0.05 |
| Skin cancer | 12 (0.01) | 4 (0.01) | 1.04 (0.34-3.23) | 0.94 |
| Stomach cancer | 146 (0.08) | 33 (0.07) | 0.91 (0.66-1.27) | 0.59 |
| Thyroid cancer | 12 (0.01) | 3 (0.01) | 0.80 (0.26-2.51) | 0.71 |
| Uterus cancer | 47 (0.05) | 18 (0.06) | 0.77 (0.48-1.22) | 0.26 |

^#^ Adjusted model: adjusted for age, gender, ethnic background, average total household income, obesity, physical activity, current smoking, alcohol intake, minerals supplementation, fruit intake, vegetable intake, processed meat intake, red meat intake, aspirin use, NSAIDS use, chondroitin use and vitamin use.
